# Prognostic rules for predicting cognitive syndromes following stroke: A systematic review

**DOI:** 10.1177/2396987321997045

**Published:** 2021-02-23

**Authors:** Bogna A Drozdowska, Kris McGill, Michael McKay, Roisin Bartlam, Peter Langhorne, Terence J Quinn

**Affiliations:** 1 Institute of Cardiovascular and Medical Sciences, University of Glasgow, UK; 2 School of Medicine, Dentistry & Nursing, University of Glasgow, UK; 3 Glasgow Royal Infirmary, National Health Service Greater Glasgow and Clyde, UK

**Keywords:** Stroke, cognition, cognitive impairment, dementia, delirium, prognosis, risk score

## Abstract

**Purpose:**

Stroke survivors are at high risk of developing cognitive syndromes, such as delirium and dementia. Accurate prediction of future cognitive outcomes may aid timely diagnosis, intervention planning, and stratification in clinical trials. We aimed to identify, describe and appraise existing multivariable prognostic rules for prediction of post-stroke cognitive status.

**Method:**

We systematically searched four electronic databases from inception to November 2019 for publications describing a method to estimate individual probability of developing a cognitive syndrome following stroke. We extracted data from selected studies using a pre-specified proforma and applied the Prediction model Risk Of Bias Assessment Tool (PROBAST) for critical appraisal.

**Findings:**

Of 17,390 titles, we included 10 studies (3143 participants), presenting the development of 11 prognostic rules – 7 for post-stroke cognitive impairment and 4 for delirium. Most commonly incorporated predictors were: demographics, imaging findings, stroke type and symptom severity. Among studies assessing predictive discrimination, the area under the receiver operating characteristic (AUROC) in apparent validation ranged from 0.80 to 0.91. The overall risk of bias for each study was high. Only one prognostic rule had been externally validated.

**Discussion/conclusion:** Research into the prognosis of cognitive outcomes following stroke is an expanding field, still at its early stages. Recommending use of specific prognostic rules is limited by the high risk of bias in all identified studies, and lack of supporting evidence from external validation. To ensure the quality of future research, investigators should adhere to current, endorsed best practice guidelines for conduct of prediction model studies.

## Introduction

Stroke survivors are at a high risk of developing cognitive syndromes, which can compromise independence in daily activities, social participation and quality of life.^1,2^ Cognitive problems can be evident at all stages of the stroke journey, from acute deficits and delirium in the early days,^3^ to persisting single and multidomain impairment,^4,5^ where for some the latter will eventually take on the severe, progressive form of post-stroke dementia.^6,7^

Change to cognitive function following stroke is a heterogeneous process, with many factors likely to affect its course.^8,9^ Accurate prognosis of cognitive syndromes is, therefore, likely to require the approach applied in prediction model studies.^10^ This entails considering a range of candidate predictor variables, identified based on existing evidence, from which those with an independent association with an outcome are selected. They are then combined to estimate the probability for an individual to develop that specific outcome, forming a prognostic rule.

A robust instrument for predicting post-stroke cognitive outcomes may improve risk stratification and inform provision of appropriate treatments and support. Many tools for predicting cognitive disorders in the general community-dwelling and inpatient population have been described.^11,12^ However, stroke cases arguably require bespoke prognostic rules that take account of distinctive baseline risk factors, the acute setting, and the importance of the index stroke and consequent treatment.

The aim of this review was to identify, describe and appraise existing prognostic rules for predicting post-stroke cognitive syndromes. Our assessment of rule performance and utility considered both the development process, and any external validation or impact studies.

## Methods

We completed this review in accordance with the Checklist for critical Appraisal and data extraction for systematic Reviews of prediction Modelling Studies (CHARMS)^13^ and Preferred Reporting for Systematic Review and Meta-Analyses (PRISMA) guidelines.^14^ The protocol can be accessed through the PROSPERO International Prospective Register of Systematic Reviews (registration number: CRD42020170428). Two researchers (BAD and KM) independently conducted all tasks involved in study selection, data extraction, and critical appraisal. Disagreements were discussed and resolved through consensus. Where an agreement could not be reached, a third, senior researcher was consulted (TJQ).

### Search strategy and selection criteria

We searched MEDLINE (OVID), EMBASE (OVID), PsycINFO (EBSCO), and CINAHL (EBSCO) from inception to November 13, 2019. Our aim was to identify studies describing the development, validation or impact of prognostic rules for prediction of post-stroke cognitive syndromes. Under the term of “post-stroke cognitive syndromes”, we encapsulated delirium as an acute (direct) consequence of stroke, and any form of global cognitive impairment, developed in the short or longer-term following stroke, including mild cognitive impairment and dementia.

We developed a search strategy based on validated search filters, tailored to the specific review question with support from a Cochrane Information Specialist. For all databases, the search involved terms relevant to stroke, cognition and prognosis, combined with the Boolean operator AND. The search was limited to human studies published in English. Additional studies were identified through screening reference lists of relevant reviews, and backward and forward citation searches from included publications. The full search strategy is available in the Supplemental Material.

The inclusion criteria were intentionally broad. We screened titles and abstracts using the Rayyan Qatar Computing Research Institute online application.^15^ Studies were eligible if they included participants aged 18 or over, with a clinical diagnosis of stroke. Relevant outcomes reflected global cognitive status, determined by the use of brief screening tools, neuropsychological batteries, or expert individual or consensus diagnosis, based on recognised medical classification criteria. Apart from case studies, all study designs were potentially eligible, provided predictor data related to an earlier time-point than the outcome. In relation to randomised controlled trials, we applied additional inclusion criteria for prognostic models to either have been developed in the control arm or to include receiving the experimental intervention as a predictor.

We excluded studies that involved participants with subarachnoid haemorrhage, predicted performance within one specific cognitive domain only (e.g. language), assessed cognitive outcome based on self-report measures, or were not available as a full published paper in a peer-reviewed journal. We applied no limits based on study setting or length of time from index stroke to outcome assessment. In the final stage, we excluded prognostic model development studies that did not provide a method for estimating individual outcome probability (e.g. using a mathematical formula, graphical tool or online calculator).

### Data extraction and quality assessment

We used a pre-specified, piloted proforma to extract data from selected studies, including information on: study setting, development sample characteristics, predictor and outcome variables, methods of model derivation and validation, and measures of prediction rule performance. We distinguished the following levels of prognostic rule validation, beginning from least stringent: 1) apparent, 2) internal, 3) temporal, and 4) external validation.^16^ Definitions of validation strategies and a description of considered prognostic rule performance measures are presented in the Supplemental Material.

We assessed risk of bias for each included study using the Prediction model Risk Of Bias Assessment Tool (PROBAST).^17^ The tool comprises four domains: participants, predictors, outcome and analysis, each appraised separately and then considered in conjunction to make an overall judgment on risk of bias (internal validity). Three study domains (with the exclusion of analysis) are additionally rated on applicability, i.e. relevance to the populations and settings targeted by the review.

## Results

**Figure 1.**
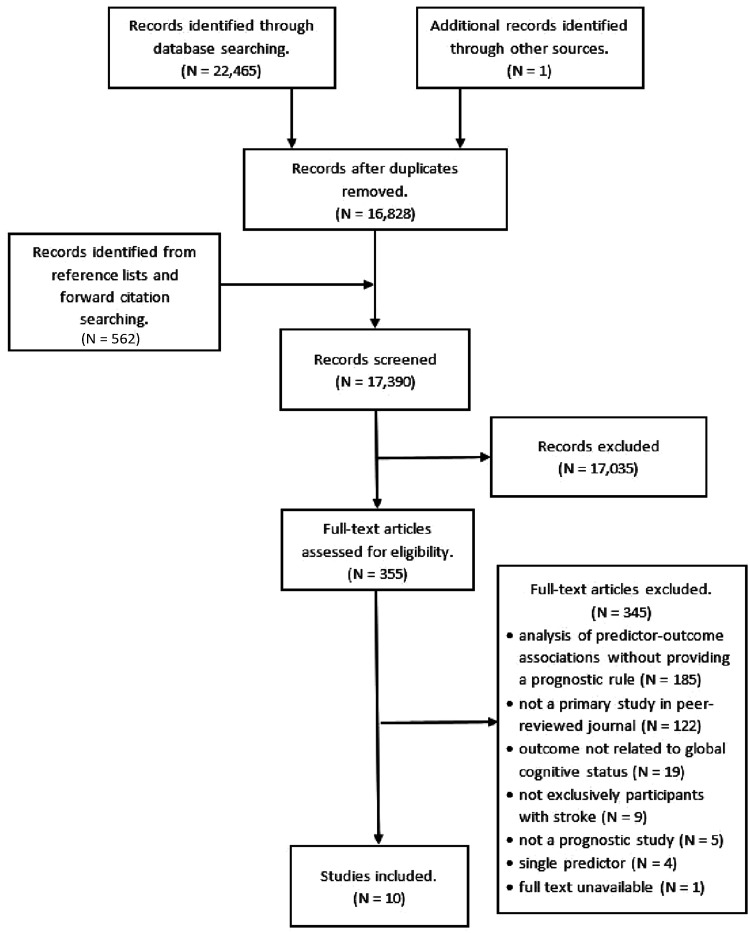
Flowchart of study selection and inclusion process.

Of 17,390 titles, we included 10 studies, presenting the development of 11 prognostic rules (Figure 1).^18–27^ In total, 3143 participants from seven different Asian and European countries were involved in development of prognostic models. Predicted post-stroke outcomes included any form of global cognitive impairment, dementia, and delirium. Due to differences in clinical course, considered risk factors, and in turn – related modelling challenges – we described prognostic rules for delirium separately. Characteristics of identified development studies are summarised in Table 1. Characteristics of included participants are presented in Supplemental Table 1.

**Table 1.** Characteristics of included studies.

Study	Country	Setting	Design	Stroke type	Development sample size, *N*
*Prognostic rules for cognitive impairment*					
Chander 2017 (CHANGE)	Singapore	Tertiary outpatient stroke clinic	Retrospective cohort	Ischaemic	209
Ding 2019	China	Neurology department of university hospital	Prospective cohort	Ischaemic	145
Gong 2019	China	Hospital rehabilitation department	Retrospective cohort	Supratentorial haemorrhage	92
Kandiah 2016 (SIGNAL_2_)	Singapore	Tertiary outpatient stroke clinic	Retrospective cohort	Ischaemic	209
Lin 2003	Taiwan	Neurology department of university hospital	Prospective cohort	Ischaemic	283
Munsch 2016	France	Neurology department of university hospital	Prospective cohort	Supratentorial ischaemia	198
Salihovic 2018	Bosnia and Herzegovina	Neurology department at a university clinical centre	Prospective cohort	Ischaemic, haemorrhagic	275
*Prognostic rules for delirium*					
Kostalova 2012	Czech Republic	Stroke unit of university hospital	Prospective cohort	Ischaemic, haemorrhagic	100
Kotfis 2019 (DELIAS)	Poland	Neurology department of district general hospital	Prospective cohort	Ischaemic	1001
Oldenbeuving 2014	Netherlands	Stroke units of two general hospitals	Prospective cohort	Ischaemic, haemorrhagic	527

### Prognostic rules for cognitive impairment

#### Overview

Seven studies addressed development of post-stroke cognitive impairment,^18–24^ including two focusing specifically on dementia (Table 2).^22,24^ The CHANGE^18^ score was developed to overcome limitations of an earlier prognostic rule – SIGNAL_2_^21^ – and was derived using the same dataset. All studies explicitly stated exclusion of individuals with pre-existing cognitive impairment, four studies excluded participants based on severity of specified symptoms,^18,19,21,24^ and four – based on psychiatric comorbidity.^18,19,21,23^ One prognostic rule was developed based on Chi-square Automatic Interaction Detection (CHAID) algorithm analysis,^24^ while all other studies employed logistic regression. On average, the identified prognostic rules included five variables (range: 3–7). Greatest overlap was observed for demographics and imaging findings, with both types of variables incorporated into five prognostic rules (Table 3). Across all studies, participant outcomes were determined at 3 to 12 months post-stroke. Among the five studies that assessed discrimination, the reported area under the receiver operating characteristic (AUROC) in apparent validation ranged from 0.81 (good)^23^ to 0.91 (excellent).^20^

**Table 2.** Prognostic rule predictors, outcomes and discriminatory power.

Study	Predictors	Outcome	Assessment	Timepoint of outcome assessment	Participants with outcome, N (%)	Validation strategy and corresponding AUROC (95% CI)
*Prognostic rules for cognitive impairment*						
Chander 2017 (CHANGE)	Age, education, acute nonlacunar cortical infarcts, chronic lacunes, white matter hyperintensities, global cortical atrophy	Cognitive impairment	Structured clinical interview and MMSE, MoCA if further confirmation required	At 3 to 6 mths post-stroke	78 (37.3%)	Apparent: 0.82 (0.76–0.88); temporal: 0.78 (0.71–0.85); external: 0.75 (0.71–0.79)
Ding 2019	Age, education, acute nonlacunar infarcts, periventricular hyperintensity, diabetes mellitus	Cognitive impairment	MMSE, MoCA, neuropsychological battery, assessment based on CDR and DSM-IV criteria	At 6 to 12 mths post-stroke	77 (53.1%)	Apparent: 0.88 (0.83–0.94)
Gong 2019	Intraventricular haemorrhage, GCS score, bleeding volume	Cognitive impairment	MMSE	At 3 to 6 mths post-stroke	69 (54.3%) of 127^a^	Apparent: 0.91 (CI not reported); internal with data splitting based on recruitment period: 0.92 (CI not reported)
Kandiah 2016 (SIGNAL_2_)	Age, education, acute nonlacunar cortical infarcts, chronic lacunes, white matter hyperintensities, global cortical atrophy, intracranial stenosis	Cognitive impairment	Structured clinical interview and MMSE, MoCA if further confirmation required	At 3 to 6 mths post-stroke	78 (37.3%)	Apparent: 0.83 (0.77–0.88); temporal: 0.78 (0.70–0.85)
Lin 2003	Age, occupation, previous stroke, vascular territory of infarction, NIHSS score, MMSE score, FIM motor score	Dementia	Consensus diagnosis; neuropsychological battery, CERAD scores, CDR; criteria: ICD-10NA, NINDS, NINDS-AIREN, and Alzheimer’s Disease and Related Disorders Association	At 3 mths post-stroke	26 (9.2%)	Apparent: 93.4% of participants correctly classified according to outcome
Munsch 2016	Age, infarct volume, NIHSS score, stroke location expressed as number of eloquent voxels from voxel-based lesion-symptom mapping maps	No cognitive impairment	MoCA	At 3 mths post-stroke	77 (38.9%)	Apparent: 0.81 (0.75–0.87); internal with 10-fold cross validation and 1000 bootstrap replications: 0.77 (0.69–0.84); internal with data splitting based on recruitment period: 0.78 (0.70-0.85)
Salihovic 2018	Complex figure test score, narrative memory score, numerical memory score	Vascular dementia	Diagnosis using clinical exams and neuropsychological testing, based on DSM-IV and ICD-10 criteria	At 12 mths post-stroke	190 (69.1%)	Not assessed
*Prognostic rules for delirium*						
Kostalova 2012; Rule 1	Age, intracerebral haemorrhage, lesion volume, gamma-glutamyl transferase, bilirubin	Delirium	Consensus diagnosis based on DSM-IV criteria, CAM-ICU	Within 24 hrs of admission, then daily for 7 days	43 (43.0%)	Internal with 2-fold cross-validation: correctly classified 69.0% of participants with delirium and 84.2% without
Kostalova 2012; Rule 2	Age, intracerebral haemorrhage, lesion volume, SOFA-Max	Delirium	Consensus diagnosis based on DSM-IV criteria, CAM-ICU	Within 24 hrs of admission, then daily for 7 days	43 (43.0%)	Internal with 2-fold cross-validation: correctly classified 65.1% of participants with delirium and 80.7% without
Kotfis 2019 (DELIAS)	Age, NIHSS score, hemianopia, aphasia, neutrophil to lymphocyte ratio, leukocytes, c-reactive protein	Early-onset delirium (24 hours), delirium up to 5 days	CAM-ICU and investigator assessment based on DSM-V criteria	At admission, then daily up to 5 days	172 (17.2%)	Apparent, for early onset delirium: 0.80 (CI not reported); for delirium up to 5 days: 0.73 (CI not reported)
Oldenbeuving 2014	Age, stroke subtype, NIHSS score, infection	Delirium	CAM	Twice after admission: between days 2 and 4, and days 5 and 7	62 (11.8%)	Apparent: 0.84 (0.80–0.89); temporal: 0.83 (0.77–0.90)

^a^Combined development and validation cohorts.

AUROC: area under the receiver operating characteristic; CAM: Confusion Assessment Method; CAM-ICU: Confusion Assessment Method for the Intensive Care Unit; CDR: Clinical Dementia Rating Scale; CERAD: Consortium to Establish a Registry for Alzheimer’s Disease; CI: confidence interval; DSM-IV and DSM-V: Diagnostic and Statistical Manual of Mental Disorders, fourth edition and fifth edition; FIM: Functional Independence Measure; GCS: Glasgow Coma Scale; hrs: hours; ICD-10NA: International Classification of Diseases, tenth revision: Neurological Adaptation; MMSE: Mini-Mental State Examination; MoCA: Montreal Cognitive Assessment; mths: months; NIHSS: National Institutes of Health Stroke Scale**;** NINDS: National Institute of Neurologic and Communicative Disorders and Stroke; NINDS-AIREN: National Institute of Neurological Disorders and Stroke–Association Internationale pour la Recherche et l’Enseignement en Neurosciences; SOFA-Max: Sequential Organ Failure Assessment, maximum score.

**Table 3.** Types of variables included in prognostic rules.

Study	Demographics	Medical history	Symptom severity	Stroke type	Imaging findings	Acute medical complications	Laboratory markers	Baseline function
*Prognostic rules for cognitive impairment*								
Chander 2017 (CHANGE)	✓				✓			
Ding 2019	✓	✓			✓			
Gong 2019			✓	✓	✓			
Kandiah 2016 (SIGNAL_2_)	✓				✓			
Lin 2003	✓	✓	✓	✓				✓
Munsch 2016	✓		✓		✓			
Salihovic 2018								✓
*Prognostic rules for delirium*								
Kostalova 2012; Rule 1	✓			✓	✓		✓	
Kostalova 2012; Rule 2	✓			✓	✓	✓		
Kotfis 2019 (DELIAS)	✓		✓				✓	✓
Oldenbeuving 2014	✓		✓	✓		✓		

#### Risk of bias and applicability

We rated all studies as high risk of bias in the domain of analysis (Table 4), with two reasons applicable to each case – inappropriate handling of missing data and not accounting for data complexities (use of analysis methods that do not allow for inclusion of censored participants). Two studies did not assess discrimination,^22,24^ while calibration was not assessed appropriately^19^ or at all^22–24^ in four studies. Assessment of rule performance was limited to apparent validation in two studies,^19,22^ while no validation procedure was reported by Salihovic et al.^24^ Only CHANGE^18^ was externally validated. Given the broad review question, applicability was overall of low concern, with one exception in the domain of predictors. Munsch et al.^23^ obtained information on stroke location based on the outcome, using lesion symptom mapping, rather than prior to outcome assessment.

**Table 4.** Assessment of risk of bias.

Study	Participants	Predictors	Outcome	Analysis	Overall assessment
*Prognostic rules for cognitive impairment*					
Chander 2017 (CHANGE)	**−**	**+**	**−**	**−**	**−**
Ding 2019	**+**	**+**	**?**	**−**	**−**
Gong 2019	**+**	**+**	**+**	**−**	**−**
Kandiah 2016 (SIGNAL_2_)	**−**	**+**	**−**	**−**	**−**
Lin 2003	**+**	**+**	**−**	**−**	**−**
Munsch 2016	**+**	**−**	**+**	**−**	**−**
Salihovic 2018	**+**	**+**	**−**	**−**	**−**
*Prognostic rules for delirium*					
Kostalova 2012	**+**	**+**	**−**	**−**	**−**
Kotfis 2019 (DELIAS)	**+**	**?**	**−**	**−**	**−**
Oldenbeuving 2014	**+**	**−**	**−**	**−**	**−**

**+** indicates low risk of bias; **−** indicates high risk of bias; ? indicates unclear risk of bias.

## Prognostic rules for delirium

### 

#### Overview

Three studies aimed to predict risk of delirium,^25–27^ producing four prognostic rules (two alternatives in Kostalova et al.^25^). None of the studies excluded individuals based on pre-existing cognitive impairment, one study excluded subjects with a history of psychiatric disorder (psychosis).^25^ All prognostic models were developed using logistic regression analysis. On average, the prognostic rules included five variables (range: 4–7). Each used demographic information, and three incorporated stroke type. When compared to rules for cognitive impairment, we found inclusion of two types of variables to be unique – acute medical complications^25,27^ and laboratory markers.^25,26^ On account of the fluctuating course of delirium, in all studies the outcome was assessed on multiple occasions. Kostalova et al.^25^ and Kotfis et al.^26^ conducted assessments daily for up to eight and six days, respectively, including the day of hospital admission. Oldenbeuving et al.^27^ screened for delirium on two separate days within a seven day period from admission. Regarding rule performance, out of the two studies that assessed discrimination in apparent validation, Oldenbeuving et al.^27^ reported the higher estimate (AUROC = 0.84).

#### Risk of bias and applicability

We rated all studies as high risk of bias in domains of outcome and analysis. Regarding the former, risk of bias was judged as high due lack of blinding to predictors, or even use of predictor knowledge to inform outcome assessment. In terms of analysis, common concerns related to insufficient sample size, inappropriate handling of missing data and/or data complexities, and no evaluation of rule calibration. Assessment of discrimination was omitted from the study by Kostalova et al.,^25^ while Kotfis et al.^26^ applied no method to adjust for optimism in estimating the performance of DELIAS. Among the three studies, the most stringent form of validation (temporal) was applied by Oldenbeuving et al.^27^

Applicability was of high concern in studies by Kostalova et al.^25^ and Oldenbeuving et al.,^27^ due to risk of overlap in timing of predictor and outcome assessments. Regarding the publication by Kotfis et al.,^26^ we rated applicability as unclear, as we could not ascertain whether predictor information was obtained prior to the outcome.

## Discussion

We identified 11 prognostic rules for prediction of post-stroke cognitive syndromes, three of which had been published in the last year. However, none of these rules are ready for routine clinical use, with no independent external validation, or assessments describing implementing the prognostic rules in practice. Research into prognosis of post-stroke cognitive outcomes is an expanding area, but still at its early stages, with a primary focus on development of novel strategies, rather than validation or application.

### Clinical implications

Based on our data, preferred prognostic rules for prediction of either post-stroke delirium or cognitive impairment cannot be recommended. All included studies had strengths and limitations. The study by Gong et al.^20^ was rated to have high risk of bias in only one domain, and reported the highest discriminatory power in apparent validation (AUROC = 0.91). However, it is important to note the small development sample size, and that the rule was developed exclusively for use in haemorrhagic stroke.

Studies by Chander et al. (CHANGE)^18^ and Oldenbeuving et al.^27^ applied the most stringent validation strategies and produced risk scores that allow for easy estimation of individual prognosis. Yet, the same two studies had the highest number of domains rated as high risk of bias (three out of four) in our quality assessment. In external validation, CHANGE^18^ was shown to have only fair discriminatory power. This measure of predictive ability was unavailable for any of the other prognostic rules.

A fundamental challenge is that without external validation studies, the generalisability of developed prognostic rules cannot be assessed. To choose an optimal prognostic rule, it is also essential to consider the target population and setting. For example, CHANGE^18^ and SIGNAL_2_^21^ were specifically intended for survivors of non-disabling stroke, and may not be applicable in an unselected stroke population. Generalisability may also me compromised if a tool relies on predictor information that is not available in all healthcare systems. An example is use of a neuroimaging score, which may not be attainable in resource-poor settings. Ultimately, uptake of prognostic rules in clinical practice is likely to depend on ease of use, clear implications of the prognostic tool results, and the economic and opportunity cost of any changes in workload.

### Research implications

Until external validation studies become available, risk of bias ratings offer some indication of how well a prognostic rule may perform when applied in a new population. Common themes from our PROBAST ratings highlight some of the challenges inherent to stroke prognostic research. Stroke-related impairments limiting completion of certain assessments,^28^ deaths and losses to follow-up can all contribute to missing data and biased study samples. Another challenge relates to incorporation bias, where the assessment of the outcome of interest requires knowledge of factors that inform the prognostic tool. For example, a diagnosis of delirium according to the Diagnostic and Statistical Manual of Mental Disorders, 5th edition (DSM-5)^29^ requires obtaining evidence of a potential cause, such as infection. Such pitfalls may be impossible to avoid, yet other identified issues could have been at least partially ameliorated, e.g. enabling inclusion of participants with missing data by applying imputation techniques.

Despite these limitations, the reviewed studies form an important foundation for future research into stroke and cognition. They identify key predictors for development of cognitive disorders, and are necessary for the next stages of prediction model research – external validation, updating and impact assessment. All with the ultimate aim of implementing useful prognostic rules in routine practice.

Recognition of methodological limitations encountered in existing publications, and application of the comprehensive, rigorous and explicit guidance presented within the recently published PROBAST tool, can help raise standards in design, conduct and reporting of future prediction model studies. The development and validation of prognostic tools requires multiple, large datasets of individual participant data and prospective follow-up. The stroke cognition community benefits from collective efforts to bring together international datasets with a cognitive focus, and these provide an ideal platform for future prognostic research.^30^

### Strengths and limitations

This is the first systematic review to focus on prognostic rules for prediction of post-stroke cognitive syndromes – a priority concern for many stroke survivors. The opportunity to use the relatively novel PROBAST tool posed an important advantage to completing this work. Lack of a consensus approach to risk of bias assessment has limited previous prognostic reviews.^31^ Another strength relates to the search strategy and inclusion criteria being specifically designed to ensure comprehensiveness.

However, due to limited resources, the search only focused on studies published in English. Moreover, by requiring that publications provide a method to estimate the individual probability of cognitive outcomes, studies relying on more complex prediction techniques, such as machine learning, would have been excluded. However, this was deliberate to ensure the review would be useful to clinicians and researchers through focusing on methods which are prepared for immediate application, provided predictor information is readily available.

## Conclusion

Research into prognosis of post-stroke cognitive outcomes is evolving. At present, any recommendation for implementing specific prognostic rules seems premature, due to high risk of bias identified in all considered studies, lack of evidence from external validation, and applicability being dependent on the context of use. Adherence to current, endorsed best practice guidelines for developing and validating prognostic rules is key to ensure improvement in the quality of future research work. External validation studies are particularly needed for this field to progress to its next stages, with a view to eventually implement prognostic rules for post-stroke cognitive outcomes in routine practice.

## Supplemental Material

sj-pdf-1-eso-10.1177_2396987321997045 - Supplemental material for Prognostic rules for predicting cognitive syndromes following stroke: A systematic review Click here for additional data file. Supplemental material, sj-pdf-1-eso-10.1177_2396987321997045 for Prognostic rules for predicting cognitive syndromes following stroke: A systematic review by Bogna A Drozdowska, Kris McGill, Michael McKay, Roisin Bartlam, Peter Langhorne and Terence J Quinn in European Stroke Journal

sj-pdf-2-eso-10.1177_2396987321997045 - Supplemental material for Prognostic rules for predicting cognitive syndromes following stroke: A systematic review Click here for additional data file. Supplemental material, sj-pdf-2-eso-10.1177_2396987321997045 for Prognostic rules for predicting cognitive syndromes following stroke: A systematic review by Bogna A Drozdowska, Kris McGill, Michael McKay, Roisin Bartlam, Peter Langhorne and Terence J Quinn in European Stroke Journal
