# Supplementary material for: Prognostic rules for predicting cognitive syndromes following stroke: A systematic review
Source: Eur Stroke J. 2021 Feb 23;6(1):18–27. doi: 10.1177/2396987321997045 (PMC7995322; doi:10.1177/2396987321997045)
Supplement: sj-pdf-2-eso-10.1177_2396987321997045 - Supplemental material for Prognostic rules for predicting cognitive syndromes following stroke: A systematic review [file sj-pdf-2-eso-10.1177_2396987321997045.pdf]

# SUPPLEMENTAL MATERIALS

# Prognostic rules for predicting cognitive syndromes following stroke: A systematic review

## Supplemental methods

### *Search strategy*

We developed the search strategy based on validated search filters for terms relevant to stroke,[1] cognition[2,3] and prognosis,[4] tailored to the specific review question with support from a Cochrane Information Specialist. For all databases, the search was limited to human studies published in English. We identified additional studies through screening reference lists of relevant reviews, screening reference lists of included publications, and forward citation searches using Google Scholar.

Medline (via OVID) search strategy:

1. cerebrovascular disorders/ OR exp basal ganglia cerebrovascular disease/ OR exp brain ischemia/ OR exp intracranial arterial diseases/ OR exp "intracranial embolism and thrombosis"/ OR exp intracranial hemorrhages/ OR stroke/ OR exp brain infarction/ OR vasospasm, intracranial/
2. (stroke OR post?stroke OR cerebrovasc$ OR brain vasc$ OR cerebral vasc$ OR cva$ OR apoplex$ OR SAH).ti,ab.
3. ((brain$ OR cerebr$ OR cerebell$ OR intracran$ OR intracerebral) adj5 (isch?emi$ OR infarct$ OR thrombo$ OR emboli$ OR occlus$)).ti,ab.
4. ((brain$ OR cerebr$ OR cerebell$ OR intracerebral OR intracranial OR subarachnoid) adj5 (h?emorrhage$ OR h?ematoma$ OR bleed$)).ti,ab.
5. ((transi$ adj3 isch?em$ adj3 attack$) OR TIA$1).ti,ab.
6. 1 OR 2 OR 3 OR 4 OR 5
7. ((validat$ OR predict$ OR prognos$ OR rule$) adj3 (outcome$ OR risk$ OR model$)).ti,ab.
8. (prognos$ AND (method$ OR history OR variable$ OR criteria OR scor$ OR characteristic$ OR finding$ OR factor$ OR model$)).ti,ab.

9. ((history OR variable$ OR criteria OR scor$ OR characteristic$ OR finding$ OR factor$) adj3 (predict$ OR model$ OR decision$ OR identif$ OR prognos$)).ti,ab.
10. (decision$ adj3 (model$ OR clinical$)).ti,ab.
11. (stratification OR discriminat$ OR calibration).ti,ab.
12. ROC curve/
13. (c-statistic OR c statistic OR area under the curve OR AUC).ti,ab.
14. (indices OR algorithm OR multivariable).ti,ab.
15. 7 OR 8 OR 9 OR 10 OR 11 OR 12 OR 13 OR 14
16. exp dementia/
17. delirium/
18. delirium, dementia, amnestic, cognitive disorders/
19. exp cognition disorders/
20. exp cognition/
21. memory/
22. dement$.ti,ab.
23. (Alzheimer$ OR AD).ti,ab.
24. deliri$.ti,ab.
25. ((cognit$ OR memory OR mental OR brain) adj3 (func$ OR perform$ OR abilit$ OR declin$ OR reduc$ OR impair$ OR disorder$ OR fail$ OR los$ OR deficit$ OR stop$ OR progress$ OR improve$)).ti,ab.
26. mental perform$.ti,ab.
27. (memory adj3 (complain$ or declin$ or function$)).ti,ab.
28. 16 OR 17 OR 18 OR 19 OR 20 OR 21 OR 22 OR 23 OR 24 OR 25 OR 26 OR 27
29. 6 AND 15 AND 28

Embase (via OVID) search strategy:

1. cerebrovascular disease/ OR exp basal ganglion hemorrhage/ OR exp brain hematoma/ OR exp brain hemorrhage/ OR exp brain infarction/ OR exp brain ischemia/ OR cerebral artery disease/ OR exp cerebrovascular accident/ OR exp occlusive cerebrovascular disease/ OR vertebrobasilar insufficiency/ OR stroke/ OR stroke patient/ OR stroke unit/

2. (stroke OR post?stroke OR cerebrovasc$ OR brain vasc$ OR cerebral vasc$ OR cva$ OR apoplex$ OR SAH).ti,ab.
3. ((brain$ OR cerebr$ OR cerebell$ OR intracran$ OR intracerebral) adj5 (isch?emi$ OR infarct$ OR thrombo$ OR emboli$ OR occlus$)).ti,ab.
4. ((brain$ OR cerebr$ OR cerebell$ OR intracerebral OR intracranial OR subarachnoid) adj5 (h?emorrhage$ OR h?ematoma$ OR bleed$)).ti,ab.
5. ((transi$ adj3 isch?em$ adj3 attack$) OR TIA$1).ti,ab.
6. 1 OR 2 OR 3 OR 4 OR 5
7. ((validat$ OR predict$ OR prognos$ OR rule$) adj3 (outcome$ OR risk$ OR model$)).ti,ab.
8. (prognos$ AND (method$ OR history OR variable$ OR criteria OR scor$ OR characteristic$ OR finding$ OR factor$ OR model$)).ti,ab.
9. ((history OR variable$ OR criteria OR scor$ OR characteristic$ OR finding$ OR factor$) adj3 (predict$ OR model$ OR decision$ OR identif$ OR prognos$)).ti,ab.
10. (decision$ adj3 (model$ OR clinical$)).ti,ab.
11. (stratification OR discriminat$ OR calibration).ti,ab.
12. receiver operating characteristic/
13. (c-statistic OR c statistic OR area under the curve OR AUC).ti,ab.
14. (indices OR algorithm OR multivariable).ti,ab.
15. 7 OR 8 OR 9 OR 10 OR 11 OR 12 OR 13 OR 14
16. exp dementia/
17. delirium/
18. exp cognitive defect/
19. exp cognition/
20. memory/
21. dement$.ti,ab.
22. (Alzheimer$ OR AD).ti,ab.
23. deliri$.ti,ab.
24. ((cognit$ OR memory OR mental OR brain) adj3 (func$ OR perform$ OR abilit$ OR declin$ OR reduc$ OR impair$ OR disorder$ OR fail$ OR los$ OR deficit$ OR stop$ OR progress$ OR improve$)).ti,ab.
25. mental perform$.ti,ab.

26. (memory adj3 (complain$ or declin$ or function$)).ti,ab.

27. 16 OR 17 OR 18 OR 19 OR 20 OR 21 OR 22 OR 23 OR 24 OR 25 OR 26

28. 6 AND 15 AND 27

PsycINFO (via EBSCO) search strategy:

S1 DE "Cerebrovascular Disorders" OR DE "Cerebral Arteriosclerosis" OR DE "Cerebral Hemorrhage" OR DE "Cerebral Ischemia" OR DE "Cerebrovascular Accidents" OR DE "Subarachnoid Hemorrhage"

S2 TI (stroke OR post#stroke OR cerebrovasc* OR "brain vasc*" OR "cerebral vasc*" OR cva* OR apoplexy OR SAH) OR AB (stroke OR post#stroke OR cerebrovasc* OR "brain vasc*" OR "cerebral vasc*" OR cva* OR apoplexy OR SAH)

S3 TI ((brain* OR cerebr* OR cerebell* OR intracran* OR intracerebral) N5 (isch#emi$ OR infarct* OR thrombo* OR emboli* OR occlus*)) OR AB ((brain* OR cerebr* OR cerebell* OR intracran* OR intracerebral) N5 (isch#emi$ OR infarct* OR thrombo* OR emboli* OR occlus*))

S4 TI ((brain* OR cerebr* OR cerebell* OR intracerebral OR intracranial OR subarachnoid) N5 (h#emorrhage* OR h#ematoma* OR bleed*)) OR AB ((brain* OR cerebr* OR cerebell* OR intracerebral OR intracranial OR subarachnoid) N5 (h#emorrhage* OR h#ematoma* OR bleed*))

S5 TI ((transi* N3 isch#em* N3 attack*) OR TIA) OR AB ((transi* N3 isch#em* N3 attack*) OR TIA)

S6 S1 OR S2 OR S3 OR S4 OR S5

S7 TI ((validat* OR predict* OR prognos* OR rule*) N3 (outcome* OR risk* OR model*)) OR AB ((validat* OR predict* OR prognos* OR rule*) N3 (outcome* OR risk* OR model*))

S8 TI (prognos* AND (method* OR history OR variable* OR criteria OR scor* OR characteristic* OR finding* OR factor* OR model*)) OR AB (prognos* AND (method* OR history OR variable* OR criteria OR scor* OR characteristic* OR finding* OR factor* OR model*))

S9 TI ((history OR variable* OR criteria OR scor* OR characteristic* OR finding* OR factor*) N3 (predict* OR model* OR decision* OR identif* OR prognos*)) OR AB ((history OR variable* OR criteria OR scor* OR characteristic* OR finding* OR factor*) N3 (predict* OR model* OR decision* OR identif* OR prognos*))

S10 TI (decision* N3 (model* OR clinical*)) OR AB (decision* N3 (model* OR clinical*))

S11 TI (stratification OR discriminat* OR calibration) OR AB (stratification OR discriminat* OR calibration)

S12 TI ("c-statistic" OR "c statistic" OR "area under the curve" OR AUC) OR AB ("c-statistic" OR "c statistic" OR "area under the curve" OR AUC)

S13 TI (indices OR algorithm OR multivariable) OR AB (indices OR algorithm OR multivariable)

S14 S7 OR S8 OR S9 OR S10 OR S11 OR S12 OR S13

S15 DE "Dementia" OR DE "Presenile Dementia" OR DE "Pseudodementia" OR DE "Semantic Dementia" OR DE "Senile Dementia" OR DE "Vascular Dementia"

S16 DE "Neurocognitive Disorders" OR DE "Delirium" OR DE "Memory Disorders" OR DE "Cognitive Impairment"

S17 DE "Memory" OR DE "Memory Decay"

S18 DE "Cognition"

S19 TI dement* OR AB dement*

S20 TI (alzheimer* OR AD) OR AB (alzheimer* OR AD)

S21 TI deliri* OR AB deliri*

S22 TI (((cognit* OR memory OR mental OR brain) N3 (func* OR perform* OR ability* OR declin* OR reduc* OR impair* OR disorder* OR fail* OR los* OR deficit* OR stop* OR progress* OR improve*))) OR AB (((cognit* OR memory OR mental OR brain) N3 (func* OR perform* OR ability* OR declin* OR reduc* OR impair* OR disorder* OR fail* OR los* OR deficit* OR stop* OR progress* OR improve*)))

S23 TI "mental perform*" OR AB "mental perform*"

S24 TI ((memory N3 (complain* or declin* or function*))) OR AB ((memory N3 (complain* or declin* or function*)))

S25 S15 OR S16 OR S17 OR S18 OR S19 OR S20 OR S21 OR S22 OR S23 OR 24

S26 S6 AND S14 AND S25

CINAHL (via EBSCO) search strategy:

S1 (MH "Cerebrovascular Disorders") OR (MH "Basal Ganglia Cerebrovascular Disease+") OR (MH "Cerebral Ischemia+") OR (MH "Intracranial Arterial Diseases+") OR (MH “Intracranial Embolism and Thrombosis+”) OR (MH “Intracranial Hemorrhage+") OR (MH “Stroke+) OR (MH “Cerebral Vasospasm”)

S2 TI (stroke OR post#stroke OR cerebrovasc* OR "brain vasc*" OR "cerebral vasc*" OR cva* OR apoplexy OR SAH) OR AB (stroke OR post#stroke OR cerebrovasc* OR "brain vasc*" OR "cerebral vasc*" OR cva* OR apoplexy OR SAH)

S3 TI ((brain* OR cerebr* OR cerebell* OR intracran* OR intracerebral) N5 (isch#emi$ OR infarct* OR thrombo* OR emboli* OR occlus*)) OR AB ((brain* OR cerebr* OR cerebell* OR intracran* OR intracerebral) N5 (isch#emi$ OR infarct* OR thrombo* OR emboli* OR occlus*))

S4 TI ((brain* OR cerebr* OR cerebell* OR intracerebral OR intracranial OR subarachnoid) N5 (h#emorrhage* OR h#ematoma* OR bleed*)) OR AB ((brain* OR cerebr* OR cerebell* OR intracerebral OR intracranial OR subarachnoid) N5 (h#emorrhage* OR h#ematoma* OR bleed*))

S5 TI ((transi* N3 isch#em* N3 attack*) OR TIA) OR AB ((transi* N3 isch#em* N3 attack*) OR TIA)

S6 S1 OR S2 OR S3 OR S4 OR S5

S7 TI ((validat* OR predict* OR prognos* OR rule*) N3 (outcome* OR risk* OR model*)) OR AB ((validat* OR predict* OR prognos* OR rule*) N3 (outcome* OR risk* OR model*))

S8 TI (prognos* AND (method* OR history OR variable* OR criteria OR scor* OR characteristic* OR finding* OR factor* OR model*)) OR AB (prognos* AND (method*

OR history OR variable* OR criteria OR scor* OR characteristic* OR finding* OR factor* OR model*))

S9 TI ((history OR variable* OR criteria OR scor* OR characteristic* OR finding* OR factor*) N3 (predict* OR model* OR decision* OR identif* OR prognos*)) OR AB ((history OR variable* OR criteria OR scor* OR characteristic* OR finding* OR factor*) N3 (predict* OR model* OR decision* OR identif* OR prognos*))

S10 TI (decision* N3 (model* OR clinical*)) OR AB (decision* N3 (model* OR clinical*))

S11 TI (stratification OR discriminat* OR calibration) OR AB (stratification OR discriminat* OR calibration)

S12 (MH "ROC Curve")

S13 TI (("c-statistic" OR "c statistic" OR "area under the curve" OR AUC)) OR AB (("c-statistic" OR "c statistic" OR "area under the curve" OR AUC))

S14 TI (indices OR algorithm OR multivariable) OR AB (indices OR algorithm OR multivariable)

S15 S7 OR S8 OR S9 OR S10 OR S11 OR S12 OR S13 OR S14

S16 (MH "Dementia+")

S17 (MH "Delirium")

S18 (MH "Delirium, Dementia, Amnestic, Cognitive Disorders")

S19 (MH "Cognition Disorders+")

S20 (MH "Cognition")

S21 (MH "Memory") OR (MH "Memory Disorders")

S22 TI dement* OR AB dement*

S23 TI (alzheimer* OR AD) OR AB (alzheimer* OR AD)

S24 TI deliri* OR AB deliri*

S25 TI (((cognit* OR memory OR mental OR brain) N3 (func* OR perform* OR ability* OR declin* OR reduc* OR impair* OR disorder* OR fail* OR los* OR deficit* OR stop* OR progress* OR improve*))) OR AB (((cognit* OR memory OR mental OR brain) N3

(func* OR perform* OR ability* OR declin* OR reduc* OR impair* OR disorder* OR fail* OR los* OR deficit* OR stop* OR progress* OR improve*)))

S26 TI "mental perform*" OR AB "mental perform*"

S27 TI ((memory N3 (complain* or declin* or function*))) OR AB ((memory N3 (complain* or declin* or function*)))

S28 S16 OR S17 OR S18 OR S19 OR S20 OR S21 OR S22 OR S23 OR S24 OR S25 OR S26 OR S27

S29 S6 AND S15 AND S28

### *Recognised validation strategies*

We distinguished the following prognostic rule validation strategies, beginning from least stringent:[5]

(i) apparent validation, where predictive ability is assessed directly in the derivation cohort;

(ii) internal validation, where the initial dataset is split or data re-use techniques are applied, such as cross-validation or bootstrapping, to quantify overfitting and adjust for optimism;

(iii) temporal validation, where performance is evaluated in subsequent participants recruited from the same center(s), independently of the original data;

(iv) external validation, where predictive ability is assessed in new and independent data, collected from a different, appropriate participant population (here, population of stroke survivors).

### *Performance measures*

We considered discrimination and calibration to be primary measures of interest, being properties that are necessary (although not sufficient) to ensure practical value of prognostic rules.[6] Discrimination refers to the ability of a prediction rule to distinguish between those who develop a certain outcome and those who do not.[7] For binary outcomes, it is commonly expressed as the area under the receiver operating characteristic curve (AUROC). According to a typically applied rule of thumb, AUROC values are interpreted as follows: 1.00 – perfect discrimination, 0.90-0.99 – excellent, 0.80-0.89 –

good, 0.70-0.79 – fair, 0.51-0.69 – poor, <0.51 – of no value/equivalent to chance.[8] Calibration relates to the level of agreement between observed and predicted outcome probabilities.

Whether prognostic rule calibration had been assessed in model development studies was accounted for in the risk of bias rating, however, only estimates of discrimination were reported in the main article. This was due to: (i) recommended assessment of calibration involving use of plots;[9] (ii) poor discrimination being sufficient to conclude that a rule will not have prognostic utility, without the need to consider its other properties.[10] In cases where discrimination had not been assessed in the primary study, we reported other available information relating to prognostic rule performance, which particularly involved classification measures.

## Supplemental results

**Supplemental Table 1.** Participant characteristics for included studies.

| Study | Age, years, mean (SD) | Women, N (%) | NIHSS, median (IQR) |
|---|---|---|---|
| Prognostic rules for cognitive impairment | | | |
| Chander 2017 | 61.7 (12.5) | 67 (32.1%) | Not reported |
| Ding 2019 | No cognitive disorder group: Mdn = 61, IQR: 48.5-69.0; cognitive disorder group: Mdn = 64, IQR: 60.0-73.0 | 42 (29.0%) | No cognitive disorder group: 3.0 (1.0-5.0); cognitive disorder group: 4.0 (2.0-7.0) |
| Gong 2019 | 57.3 (12.2) | 28 (30.4%) | Not reported |
| Kandiah 2016 ($SIGNAL_2$) | 61.7 (12.5) | 67 (32.1%) | Not reported |
| Lin 2003 | 64.4 (8.4) | 95 (33.6%) | M = 3.6, SD = 3.1 |
| Munsch 2016 | No cognitive disorder group: Mdn = 60, range: 29-84; cognitive disorder | 77 (35.8%)/215[a] | No cognitive disorder group: 3, range: 1-10; cognitive |

| | | | |
|---|---|---|---|
| | group: Mdn = 69, range: 34-95 | | disorder group: 4.0, range: 1-25 |
| Salihovic 2018 | Females: 66.3 (2.0); Males: 65.1 (1.5) | 103 (37.5%) | Score of 0 - 7: 163 subjects (59.3%); score of 8 - 14: 89 (32.4%); score > 14: 23 (8.4%) |
| Prognostic rules for delirium | | | |
| Kostalova 2012 | 73.5 (11.5) | 47 (47.0%) | No cognitive disorder group: 9.0, 5th-95th percentile range: 4-17; cognitive disorder group: 11.0, 5th-95th percentile range: 5.0-16.0 |
| Kotfis 2019 (DELIAS) | Mdn = 71.0, IQR: 64.0-82.0 | 478 (47.8%) | No cognitive disorder group: 8.0 (4.0-14.0); cognitive disorder group: 18.0, (12.0-21.5) |
| Oldenbeuving 2014 | 72.0, range: 29-96 | 239 (45.4%) | 5.0, range: 0-36 |

[a]Sample before excluding subjects with no outcome data.

IQR: interquartile range; M: mean; Mdn: median; NIHSS: National Institutes of Health Stroke Scale; SD: standard deviation.

## Supplemental references


1. Cochrane Stroke. Search methods for the cochrane stroke group specialised register. Resources for authors and editors of reviews, https://apps.ccbs.ed.ac.uk/csrg/entity/searchmethods.pdf (accessed 7 October 2019).
2. National Institute for Health and Care Excellence (NICE). Appendix D: Review search strategies. Dementia: assessment, management and support for people living with dementia and their carers, https://www.nice.org.uk/guidance/ng97/documents/search-strategies (2018, accessed 7 October 2019).
3. Flodgren GM and Berg RC. Primary and secondary prevention interventions for cognitive decline and dementia. [Primær- og sekundærforebyggende tiltak for kognitiv svikt og demens] Rapport −2016. Oslo: Folkehelseinstituttet, 2016.
4. Geersing GJ, Bouwmeester W, Zuithoff P, et al. Search filters for finding prognostic and diagnostic prediction studies in Medline to enhance systematic reviews. *PloS One* 2012; 7: e32844. doi: 10.1371/journal.pone.0032844
5. Altman DG and Royston P. What do we mean by validating a prognostic model? *Statist Med* 2000; 19: 453-473. doi: 10.1002/(SICI)1097-0258(20000229)19:4<453::AID-SIM350>3.0.CO;2-5
6. Moons KG, de Groot JA, Bouwmeester W, et al. Critical appraisal and data extraction for systematic reviews of prediction modelling studies: The CHARMS checklist. *PLoS Med* 2014; 11: e1001744. doi: 10.1371/journal.pmed.1001744
7. Pencina MJ and D'Agostino RB. Evaluating discrimination of risk prediction models: The C statistic. *JAMA* 2015; 314: 1063–1064. doi: 10.1001/jama.2015.11082
8. Carter JV, Pan JM, Rai SN, et al. ROC-ing along: Evaluation and interpretation of receiver operating characteristic curves. *Surgery* 2016; 159: 1638–1645. doi: 10.1016/j.surg.2015.12.029
9. Steyerberg EW, Vickers AJ, Cook NR, et al. Assessing the performance of prediction models: A framework for some traditional and novel measures. *Epidemiology* 2010; 21: 128–138. doi: 10.1097/EDE.0b013e3181c30fb2

10. Alba AC, Agoritsas T, Walsh M, et al. Discrimination and calibration of clinical prediction models: Users' guides to the medical literature. *JAMA* 2017; 318: 1377-1384. doi: 10.1001/jama.2017.12126
